# (2,2′-Bipyrimidine-κ^2^ *N* ^1^,*N* ^1′^)diiodidopalladium(II)

**DOI:** 10.1107/S1600536812028292

**Published:** 2012-06-30

**Authors:** Kwang Ha

**Affiliations:** a School of Applied Chemical Engineering, Research Institute of Catalysis, Chonnam National University, Gwangju 500-757, Republic of Korea

## Abstract

In the title complex, [PdI_2_(C_8_H_6_N_4_)], the Pd^II^ ion is four-coordinated in a slightly distorted square-planar environment defined by two pyrimidine N atoms derived from a chelating 2,2′-bipyrimidine (bpym) ligand and two mutually *cis* iodide anions. The nearly planar bpym ligand [maximum deviation = 0.053 (3) Å] is slightly inclined to the least-squares plane of the PdI_2_N_2_ unit [maximum deviation = 0.055 (2) Å], making a dihedral angle of 3.9 (2)°. In the crystal, pairs of complex mol­ecules are assembled by inter­molecular C—H⋯N hydrogen bonds into dimers. Intra­molecular C—H⋯I hydrogen bonds are also observed.

## Related literature

For the crystal structure of the related Pt^II^ complex [PtI_2_(bpym)], see: Ha (2010 ▶).
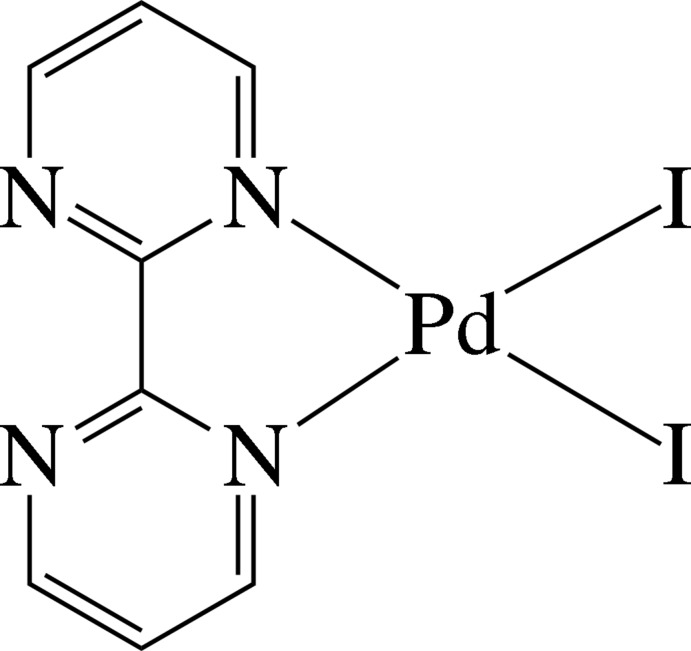


## Experimental

### 

#### Crystal data

[PdI_2_(C_8_H_6_N_4_)]
*M* *_r_* = 518.37
Monoclinic, 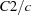

*a* = 16.1967 (10) Å
*b* = 15.2274 (10) Å
*c* = 10.4686 (6) Å
β = 113.199 (1)°
*V* = 2373.1 (3) Å^3^
*Z* = 8
Mo *K*α radiation
μ = 6.74 mm^−1^
*T* = 273 K
0.34 × 0.16 × 0.14 mm

#### Data collection

Bruker SMART 1000 CCD diffractometer
Absorption correction: multi-scan (*SADABS*; Bruker, 2000 ▶) *T*_min_ = 0.716, *T*_max_ = 1.000
7042 measured reflections
2268 independent reflections
1937 reflections with *I* > 2σ(*I*)
*R*_int_ = 0.024

#### Refinement

*R*[*F* ^2^ > 2σ(*F* ^2^)] = 0.021
*wR*(*F* ^2^) = 0.053
*S* = 1.10
2268 reflections
136 parameters
H-atom parameters constrained
Δρ_max_ = 0.64 e Å^−3^
Δρ_min_ = −0.53 e Å^−3^

### 

Data collection: *SMART* (Bruker, 2000 ▶); cell refinement: *SAINT* (Bruker, 2000 ▶); data reduction: *SAINT*; program(s) used to solve structure: *SHELXS97* (Sheldrick, 2008 ▶); program(s) used to refine structure: *SHELXL97* (Sheldrick, 2008 ▶); molecular graphics: *ORTEP-3* (Farrugia, 1997 ▶) and *PLATON* (Spek, 2009 ▶); software used to prepare material for publication: *SHELXL97*.

## Supplementary Material

Crystal structure: contains datablock(s) global, I. DOI: 10.1107/S1600536812028292/zq2173sup1.cif

Structure factors: contains datablock(s) I. DOI: 10.1107/S1600536812028292/zq2173Isup2.hkl

Additional supplementary materials:  crystallographic information; 3D view; checkCIF report

## Figures and Tables

**Table:** 

Pd1—N1	2.082 (3)
Pd1—N4	2.082 (4)
Pd1—I1	2.5696 (4)
Pd1—I2	2.5746 (5)

**Table:** 

N1—Pd1—N4	79.57 (13)
I1—Pd1—I2	89.215 (14)

**Table 2:** Hydrogen-bond geometry (Å, °)

*D*—H⋯*A*	*D*—H	H⋯*A*	*D*⋯*A*	*D*—H⋯*A*
C1—H1⋯I2	0.93	2.92	3.525 (4)	124
C8—H8⋯I1	0.93	2.91	3.522 (5)	124
C6—H6⋯N2^i^	0.93	2.60	3.523 (6)	173

Symmetry code: (i) 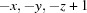.

**Fig. 1.**
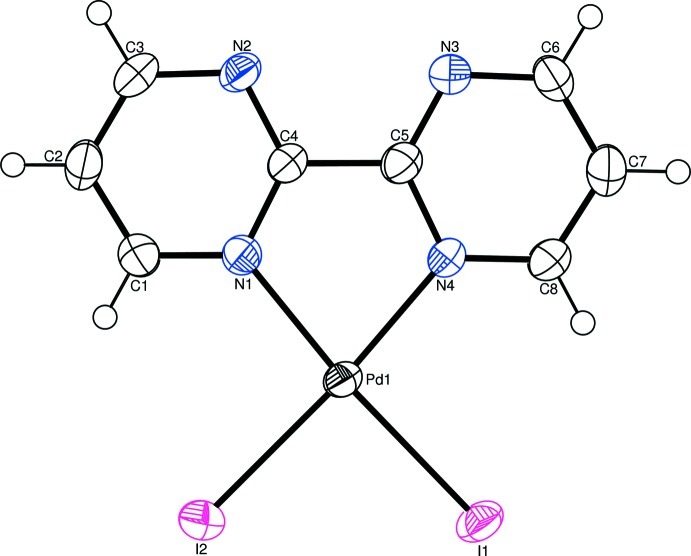
The molecular structure of the title complex, with atom numbering. Displacement ellipsoids are drawn at the 50% probability level for non-H atoms.

**Fig. 2.**
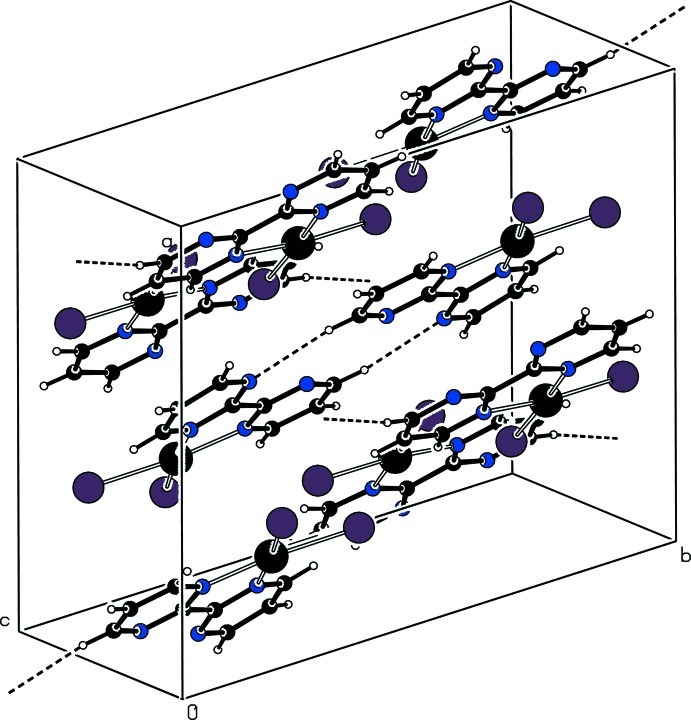
A view of the crystal packing of the title complex. Intermolecular C—H···N hydrogen-bonds are shown as dashed lines.

## supplementary crystallographic information

### Comment

The title complex, [PdI_2_(bpym)] (bpym = 2,2'-bipyrimidine, C_8_H_6_N_4_), is a structural isomer of the previously reported Pt^II^ complex [PtI_2_(bpym)] (Ha, 2010).

The Pd^II^ ion is four-coordinated in a slightly distorted square-planar environment defined by two pyrimidine N atoms derived from a chelating bpym ligand and two mutually *cis* iodide anions (Fig. 1). The main contribution to the distortion is the tight N1—Pd1—N4 chelate angle of 79.57 (13)°, which results in non-linear *trans* axes [<I1—Pd1—N1 = 175.12 (10)° and <I2—Pd1—N4 = 173.70 (9)°]. The Pd—N and Pd—I bond lengths are almost equivalent, respectively (Table 1). The nearly planar bpym ligand [maximum deviation = 0.053 (3) Å] is slightly inclined to the least-squares plane of the PdI_2_N_2_ unit [maximum deviation = 0.055 (2) Å], making a dihedral angle of 3.9 (2)°. In the crystal, two complex molecules are assembled by intermolecular C—H···N hydrogen bonds with C···N = 3.523 (6) Å, forming a dimer-type species (Fig. 2, Table 2). Intramolecular C—H···I hydrogen bonds are also observed (Table 2). The molecules are stacked in columns along the *a* axis with Pd···Pd distances of 3.8061 (5) Å and 4.6600 (6) Å. In the columns, numerous intermolecular π-π interactions between adjacent pyrimidine rings are present, the shortest ring centroid-centroid distance being 3.560 (3) Å.

### Experimental

To a solution of Na_2_PdCl_4_ (0.1472 g, 0.500 mmol) and KI (0.8381 g, 5.049 mmol) in H_2_O (20 ml) was added 2,2'-bipyrimidine (0.0792 g, 0.501 mmol), and the mixture was stirred for 3 h at room temperature. The precipitate was then separated by filtration, washed with H_2_O and acetone, and dried at 323 K, to give a redbrown powder (0.2003 g). Crystals suitable for X-ray analysis were obtained by slow evaporation from a CH_3_CN solution at room temperature.

### Refinement

H atoms were included in calculated positions and treated as riding atoms: C—H = 0.93 Å with *U*_iso_(H) = 1.2*U*_eq_(C). The highest peak (0.64 e Å^-3^) and the deepest hole (-0.53 e Å^-3^) in the difference Fourier map are located 1.10 Å and 0.93 Å, respectively, from the atoms I1 and Pd1.

### Crystal data

**Table:**

[PdI_2_(C_8_H_6_N_4_)]	*F*(000) = 1872
*M**_r_* = 518.37	*D*_x_ = 2.902 Mg m^−^^3^
Monoclinic, *C*2/*c*	Mo *K*α radiation, λ = 0.71073 Å
Hall symbol: -C 2yc	Cell parameters from 4768 reflections
*a* = 16.1967 (10) Å	θ = 2.4–26.0°
*b* = 15.2274 (10) Å	µ = 6.74 mm^−^^1^
*c* = 10.4686 (6) Å	*T* = 273 K
β = 113.199 (1)°	Block, brown
*V* = 2373.1 (3) Å^3^	0.34 × 0.16 × 0.14 mm
*Z* = 8	

### Data collection

**Table:**

Bruker SMART 1000 CCD diffractometer	2268 independent reflections
Radiation source: fine-focus sealed tube	1937 reflections with *I* > 2σ(*I*)
Graphite monochromator	*R*_int_ = 0.024
φ and ω scans	θ_max_ = 26.0°, θ_min_ = 1.9°
Absorption correction: multi-scan (*SADABS*; Bruker, 2000)	*h* = −19→19
*T*_min_ = 0.716, *T*_max_ = 1.000	*k* = −18→14
7042 measured reflections	*l* = −12→12

### Refinement

**Table:**

Refinement on *F*^2^	Primary atom site location: structure-invariant direct methods
Least-squares matrix: full	Secondary atom site location: difference Fourier map
*R*[*F*^2^ > 2σ(*F*^2^)] = 0.021	Hydrogen site location: inferred from neighbouring sites
*wR*(*F*^2^) = 0.053	H-atom parameters constrained
*S* = 1.10	*w* = 1/[σ^2^(*F*_o_^2^) + (0.0213*P*)^2^ + 1.3937*P*] where *P* = (*F*_o_^2^ + 2*F*_c_^2^)/3
2268 reflections	(Δ/σ)_max_ < 0.001
136 parameters	Δρ_max_ = 0.64 e Å^−^^3^
0 restraints	Δρ_min_ = −0.53 e Å^−^^3^

### Special details

**Table:**

Geometry. All e.s.d.'s (except the e.s.d. in the dihedral angle between two l.s. planes) are estimated using the full covariance matrix. The cell e.s.d.'s are taken into account individually in the estimation of e.s.d.'s in distances, angles and torsion angles; correlations between e.s.d.'s in cell parameters are only used when they are defined by crystal symmetry. An approximate (isotropic) treatment of cell e.s.d.'s is used for estimating e.s.d.'s involving l.s. planes.
Refinement. Refinement of *F*^2^ against ALL reflections. The weighted *R*-factor *wR* and goodness of fit *S* are based on *F*^2^, conventional *R*-factors *R* are based on *F*, with *F* set to zero for negative *F*^2^. The threshold expression of *F*^2^ > σ(*F*^2^) is used only for calculating *R*-factors(gt) *etc*. and is not relevant to the choice of reflections for refinement. *R*-factors based on *F*^2^ are statistically about twice as large as those based on *F*, and *R*- factors based on ALL data will be even larger.

### Fractional atomic coordinates and isotropic or equivalent isotropic displacement parameters (Å^2^)

**Table:**

	*x*	*y*	*z*	*U*_iso_*/*U*_eq_	
Pd1	0.15635 (2)	0.29183 (2)	0.33518 (3)	0.02688 (10)	
I1	0.254992 (19)	0.26771 (2)	0.19695 (3)	0.03728 (10)	
I2	0.16606 (2)	0.45868 (2)	0.30377 (3)	0.04108 (11)	
N1	0.0758 (2)	0.2998 (2)	0.4483 (3)	0.0281 (8)	
N2	−0.0087 (2)	0.2101 (2)	0.5372 (3)	0.0319 (8)	
N3	0.0566 (3)	0.0658 (3)	0.4549 (4)	0.0406 (9)	
N4	0.1337 (2)	0.1591 (2)	0.3566 (3)	0.0290 (8)	
C1	0.0498 (3)	0.3714 (3)	0.4974 (4)	0.0365 (11)	
H1	0.0702	0.4264	0.4846	0.044*	
C2	−0.0068 (3)	0.3644 (3)	0.5664 (4)	0.0372 (11)	
H2	−0.0241	0.4137	0.6020	0.045*	
C3	−0.0364 (3)	0.2830 (3)	0.5807 (4)	0.0359 (11)	
H3	−0.0775	0.2777	0.6222	0.043*	
C4	0.0465 (3)	0.2224 (3)	0.4733 (4)	0.0279 (9)	
C5	0.0804 (3)	0.1433 (3)	0.4274 (4)	0.0308 (10)	
C6	0.0876 (3)	−0.0035 (3)	0.4089 (5)	0.0458 (12)	
H6	0.0726	−0.0596	0.4276	0.055*	
C7	0.1411 (3)	0.0054 (3)	0.3348 (5)	0.0474 (13)	
H7	0.1613	−0.0434	0.3021	0.057*	
C8	0.1636 (3)	0.0892 (3)	0.3112 (4)	0.0360 (11)	
H8	0.2003	0.0972	0.2626	0.043*	

### Atomic displacement parameters (Å^2^)

**Table:**

	*U*^11^	*U*^22^	*U*^33^	*U*^12^	*U*^13^	*U*^23^
Pd1	0.02714 (18)	0.0290 (2)	0.02898 (18)	0.00127 (14)	0.01582 (14)	0.00223 (14)
I1	0.03387 (18)	0.0462 (2)	0.04086 (18)	0.00306 (14)	0.02450 (14)	0.00367 (14)
I2	0.0467 (2)	0.03180 (19)	0.0551 (2)	0.00018 (14)	0.03117 (16)	0.00690 (14)
N1	0.0290 (18)	0.028 (2)	0.0278 (18)	0.0023 (16)	0.0118 (15)	0.0021 (16)
N2	0.0286 (19)	0.036 (2)	0.037 (2)	−0.0042 (17)	0.0197 (16)	−0.0057 (17)
N3	0.045 (2)	0.031 (2)	0.058 (2)	−0.0066 (18)	0.034 (2)	−0.0061 (19)
N4	0.0284 (18)	0.029 (2)	0.0338 (18)	0.0016 (16)	0.0174 (15)	−0.0006 (16)
C1	0.046 (3)	0.029 (3)	0.039 (2)	0.000 (2)	0.022 (2)	0.000 (2)
C2	0.040 (3)	0.036 (3)	0.040 (3)	0.009 (2)	0.020 (2)	−0.003 (2)
C3	0.031 (2)	0.047 (3)	0.037 (2)	0.002 (2)	0.021 (2)	−0.003 (2)
C4	0.026 (2)	0.032 (3)	0.028 (2)	−0.0003 (19)	0.0129 (18)	−0.0017 (19)
C5	0.028 (2)	0.034 (3)	0.032 (2)	0.000 (2)	0.0140 (18)	−0.001 (2)
C6	0.050 (3)	0.024 (3)	0.074 (3)	−0.003 (2)	0.037 (3)	−0.006 (3)
C7	0.053 (3)	0.036 (3)	0.065 (3)	0.000 (2)	0.035 (3)	−0.011 (3)
C8	0.035 (2)	0.036 (3)	0.044 (3)	0.002 (2)	0.024 (2)	0.001 (2)

### Geometric parameters (Å, º)

**Table:**

Pd1—N1	2.082 (3)	C1—C2	1.376 (6)
Pd1—N4	2.082 (4)	C1—H1	0.9300
Pd1—I1	2.5696 (4)	C2—C3	1.358 (6)
Pd1—I2	2.5746 (5)	C2—H2	0.9300
N1—C4	1.335 (5)	C3—H3	0.9300
N1—C1	1.342 (5)	C4—C5	1.479 (6)
N2—C4	1.325 (5)	C6—C7	1.379 (6)
N2—C3	1.343 (6)	C6—H6	0.9300
N3—C5	1.309 (6)	C7—C8	1.376 (7)
N3—C6	1.338 (6)	C7—H7	0.9300
N4—C8	1.332 (6)	C8—H8	0.9300
N4—C5	1.363 (5)		
			
N1—Pd1—N4	79.57 (13)	C1—C2—H2	121.1
N1—Pd1—I1	175.12 (10)	N2—C3—C2	122.5 (4)
N4—Pd1—I1	95.56 (9)	N2—C3—H3	118.7
N1—Pd1—I2	95.66 (10)	C2—C3—H3	118.7
N4—Pd1—I2	173.70 (9)	N2—C4—N1	126.0 (4)
I1—Pd1—I2	89.215 (14)	N2—C4—C5	117.3 (4)
C4—N1—C1	116.9 (3)	N1—C4—C5	116.7 (3)
C4—N1—Pd1	114.3 (3)	N3—C5—N4	125.7 (4)
C1—N1—Pd1	128.8 (3)	N3—C5—C4	118.9 (4)
C4—N2—C3	115.9 (4)	N4—C5—C4	115.4 (4)
C5—N3—C6	116.6 (4)	N3—C6—C7	122.3 (5)
C8—N4—C5	116.8 (4)	N3—C6—H6	118.9
C8—N4—Pd1	129.3 (3)	C7—C6—H6	118.9
C5—N4—Pd1	113.9 (3)	C8—C7—C6	117.5 (5)
N1—C1—C2	120.9 (4)	C8—C7—H7	121.3
N1—C1—H1	119.6	C6—C7—H7	121.3
C2—C1—H1	119.6	N4—C8—C7	121.2 (4)
C3—C2—C1	117.8 (4)	N4—C8—H8	119.4
C3—C2—H2	121.1	C7—C8—H8	119.4
			
N4—Pd1—N1—C4	−2.9 (3)	C1—N1—C4—C5	−176.0 (3)
I2—Pd1—N1—C4	173.0 (3)	Pd1—N1—C4—C5	4.5 (5)
N4—Pd1—N1—C1	177.6 (4)	C6—N3—C5—N4	0.0 (7)
I2—Pd1—N1—C1	−6.5 (4)	C6—N3—C5—C4	178.8 (4)
N1—Pd1—N4—C8	−179.4 (4)	C8—N4—C5—N3	0.3 (6)
I1—Pd1—N4—C8	0.4 (4)	Pd1—N4—C5—N3	−179.8 (4)
N1—Pd1—N4—C5	0.7 (3)	C8—N4—C5—C4	−178.5 (4)
I1—Pd1—N4—C5	−179.5 (3)	Pd1—N4—C5—C4	1.4 (4)
C4—N1—C1—C2	−1.9 (6)	N2—C4—C5—N3	−1.8 (6)
Pd1—N1—C1—C2	177.6 (3)	N1—C4—C5—N3	177.1 (4)
N1—C1—C2—C3	−1.2 (7)	N2—C4—C5—N4	177.2 (4)
C4—N2—C3—C2	−3.0 (6)	N1—C4—C5—N4	−3.9 (6)
C1—C2—C3—N2	3.8 (7)	C5—N3—C6—C7	−0.7 (7)
C3—N2—C4—N1	−0.4 (6)	N3—C6—C7—C8	1.2 (8)
C3—N2—C4—C5	178.4 (4)	C5—N4—C8—C7	0.2 (6)
C1—N1—C4—N2	2.8 (6)	Pd1—N4—C8—C7	−179.7 (3)
Pd1—N1—C4—N2	−176.7 (3)	C6—C7—C8—N4	−0.9 (7)

### Hydrogen-bond geometry (Å, º)

**Table:**

*D*—H···*A*	*D*—H	H···*A*	*D*···*A*	*D*—H···*A*
C1—H1···I2	0.93	2.92	3.525 (4)	124
C8—H8···I1	0.93	2.91	3.522 (5)	124
C6—H6···N2^i^	0.93	2.60	3.523 (6)	173

Symmetry code: (i) −*x*, −*y*, −*z*+1.
